# Data on the DNA damaging and mutagenic potential of the BH3-mimetics ABT-263/Navitoclax and TW-37

**DOI:** 10.1016/j.dib.2016.01.013

**Published:** 2016-01-16

**Authors:** Maja M. Green, Tanmay M. Shekhar, Christine J. Hawkins

**Affiliations:** Department of Biochemistry and Genetics, La Trobe Institute for Molecular Science, La Trobe University, Melbourne, Victoria 3086, Australia

## Abstract

Unfortunately, the mutagenic activities of chemotherapy and radiotherapy can provoke development of therapy-induced malignancies in cancer survivors. Non-mutagenic anti-cancer therapies may be less likely to trigger subsequent malignant neoplasms. Here we present data regarding the DNA damaging and mutagenic potential of two drugs that antagonize proteins within the Bcl-2 family: ABT-263/Navitoclax and TW-37. Our data reveal that concentrations of these agents that stimulated Bax/Bak-dependent signaling provoked little DNA damage and failed to trigger mutations in surviving cells. The data supplied in this article is related to the research work entitled "Inhibition of Bcl-2 or IAP proteins does not provoke mutations in surviving cells" [1].

**Specifications table**

**Table:** 

Subject area	*Biology*
More specific subject area	*Mutagenesis, cancer biology, apoptosis research*
Type of data	*Graphs*
How data was acquired	*Clonogenicity assays, HPRT mutagenesis assays, γH2AX flow cytometry quantitation*
Data format	*Normalized data*
Experimental factors	*Murine embryonic fibroblasts from wild type or Bax*^−^^*/*^^−^*, Bak*^−^^*/−*^*mice were treated with various concentrations of ABT-263 or TW-37, prior to the assays listed above.*
Experimental features	*Cells were exposed to drugs (or not) for various periods of time, then washed and either stained with anti-γH2AX for flow cytometry, seeded into normal media to quantitate clonogenicity, or seeded into media containing 6-thioguanine to count 6-thioguanine-resistant clones (presumably reflecting mutagenesis at the HPRT locus).*
Data source location	*La Trobe University, Bundoora, Australia*
Data accessibility	*Data is included within this article*

**Value of the data**

• These data can be used to compare the mutagenic potentials of anti-cancer drugs that employ different mechanisms of action
• Future research could define the pathways through which high concentrations of some BH3-mimetics kill cells and damage DNA
• Researchers could use this data to design animal-based experiments to evaluate the mutagenic and oncogenic activity of Bcl-2 antagonists in vivo

## 1 Data

**Fig. 1:**
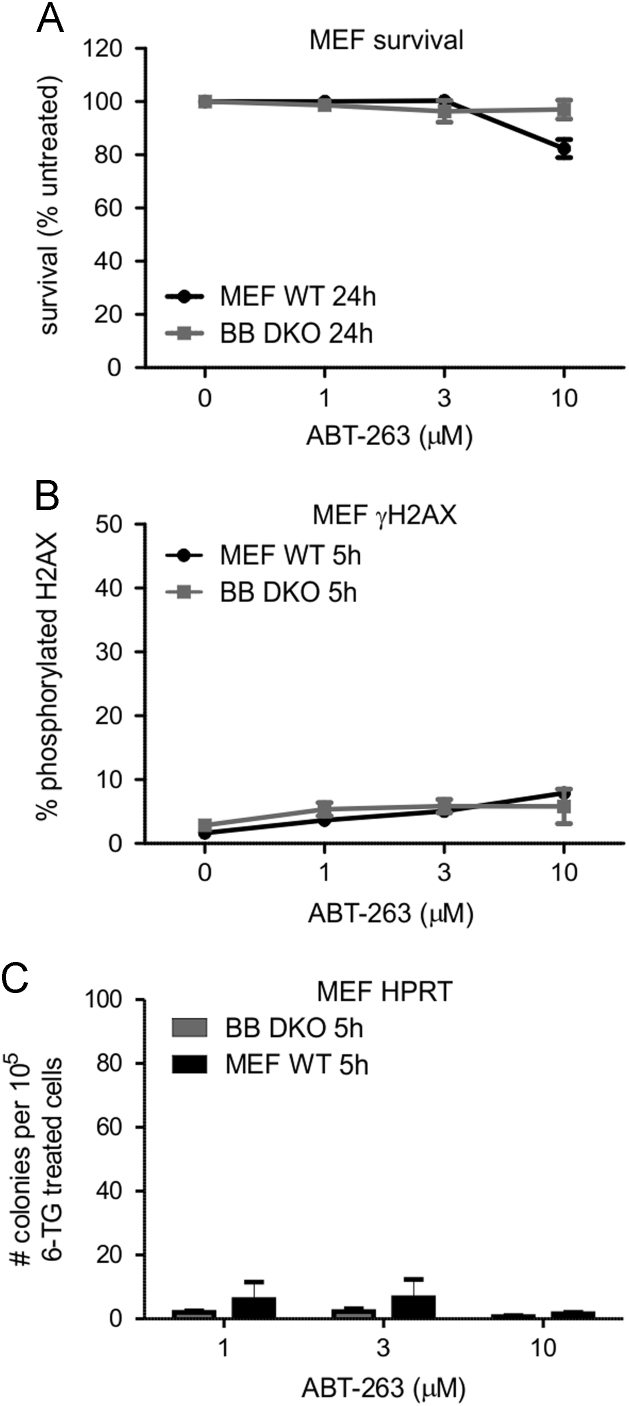
ABT-263 kills MEF cells very inefficiently and fails to stimulate DNA damage or mutagenesis in surviving cells. Murine embryonic fibroblasts (MEF) from wild type (WT) or Bax/Bak knockout (BB DKO) mice were incubated with the indicated doses of ABT-263 for the specified periods of time. The cells were then subjected to clonogenicity assays (A), analyzed by flow cytometry to determine the proportion in which H2AX proteins were phosphorylated (γH2AX) (B) or incubated in 6-TG-containing medium (C). The reported peak plasma concentration for patients administered ABT-263 was 3.6 µM [7]. (A–C) Error bars indicate standard errors of the means from three independent experiments.

**Fig. 2:**
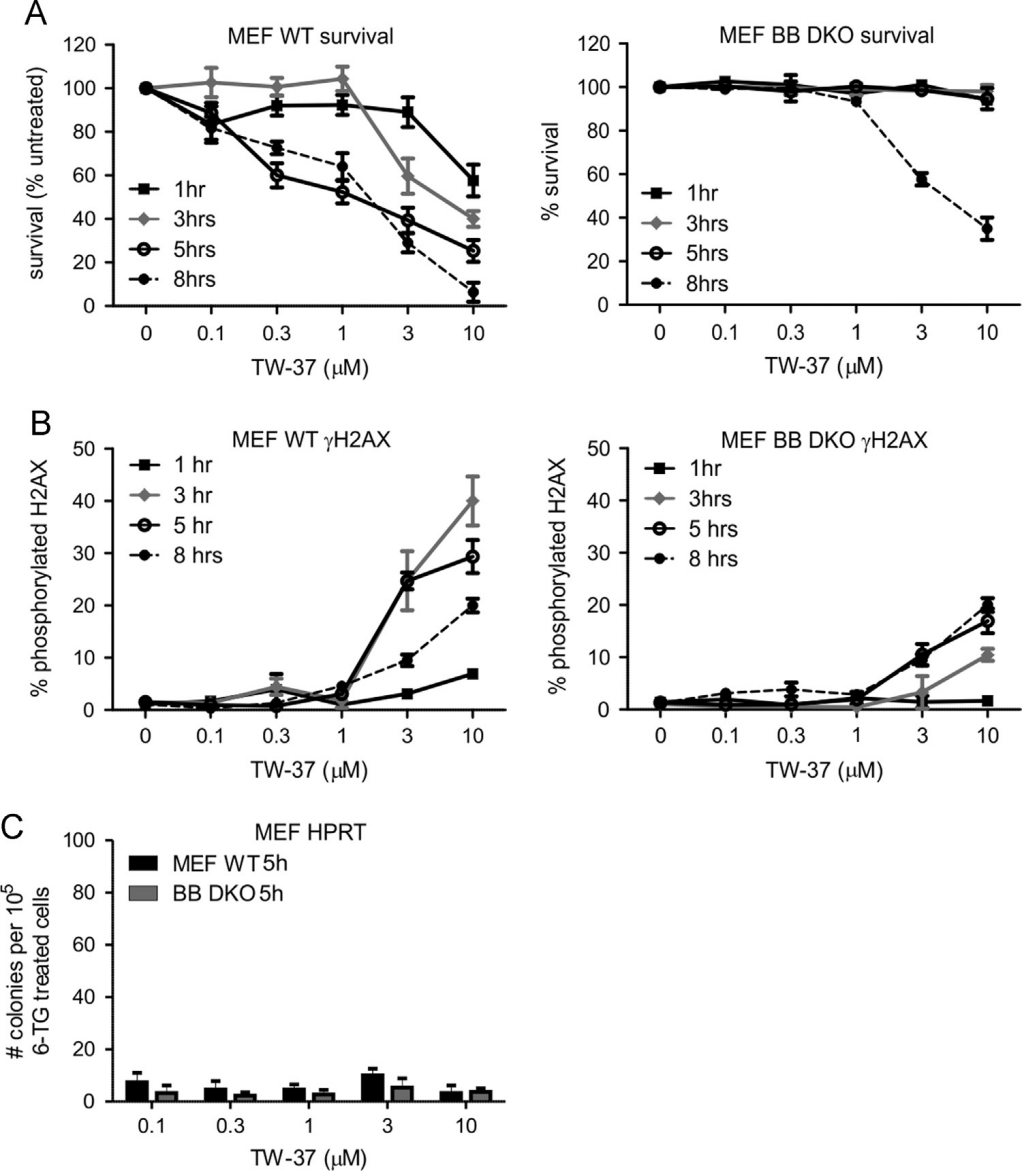
TW-37 is non-mutagenic and high concentrations damage DNA predominantly through Bax/Bak-independent pathways. Murine embryonic fibroblasts (MEF) from wild type (WT) or Bax/Bak knockout (BB DKO) mice were incubated with the indicated doses of TW-37 for the specified periods of time. The cells were then subjected to clonogenicity assays (A), analyzed by flow cytometry to determine the proportion in which H2AX proteins were phosphorylated (γH2AX) (B) or incubated in 6-TG-containing medium (C). (A–C) Error bars indicate standard errors of the means from three independent experiments.

Embryonic fibroblasts derived from wildtype or Bax/Bak-deficient mice were treated with ABT-263 (Fig. 1) or TW-37 (Fig. 2), or incubated in normal medium. We measured the impact of these treatments on survival, DNA damage and mutagenicity at the HPRT locus.

## 2 Experimental design, materials and methods

### 2.1 Cell lines and materials

SV-40 transformed Mouse Embryonic Fibroblasts (MEF) were kindly provided by Anissa Jabbour and Paul Ekert [2] and were cultured in DMEM high glucose (Invitrogen; Carlsbad, California, USA) containing 10% fetal calf serum (Invitrogen). ABT-263 and TW-37 were purchased from Selleck Chemicals (Houston, Texas, USA). The following antibodies were used: anti-H2AX (Ser 139) clone 20E3 (Cell Signaling Technology) and goat anti-rabbit FITC (Chemicon).

### 2.2 Cell survival assays

The toxicity of the drugs was assessed by comparing the clonogenic survival of treated and untreated cells. Cells were incubated with drugs, then washed and seeded at various densities in 6-well plates. After seven days, cells were stained with methylene blue (Sigma Aldrich) 1.25 g/l in 50% methanol, incubated for 5 min and washed twice with water, then the numbers of colonies were counted.

### 2.3 HPRT assay

To evaluate the ability of the drugs to provoke mutations in clonogenically-competent cells, we quantitated the emergence of colonies in media containing 6-thioguanine (6-TG), using a previously published method [1], [3]. This purine analog is toxic to cells expressing functional hypoxanthine-guanine phosphoribosyltransferase (HPRT) and growth of colonies in 6-TG following drug treatment usually results from drug-induced loss-of-function mutations at the HPRT locus [4]. Cells were incubated with the drug or normal media, then washed and incubated in normal media for eight days to allow for expression of the HPRT mutant phenotype. Cells were then seeded at 10^5^ cells per 150 mm dish (three dishes per treatment) in media containing 6-thioguanine (Sigma Aldrich). Colonies were stained with methylene blue and counted after 13 days. The number of 6-TG-resistant colonies following drug treatment was calculated by subtracting the number of background colonies that arose in untreated samples.

### 2.4 DNA damage assay

Drug-induced DNA damage was examined by measuring the proportion of cells bearing phosphorylated H2AX, which typically occurs in response to double-stranded DNA breaks [5]. Staining of phosphorylated H2AX was based on a published protocol [6]. Cells were exposed to drugs then stained using anti-phosphorylated H2AX and anti-rabbit FITC antibodies. After washing to remove unbound antibodies, the cells were resuspended in PBS containing PI (5 µg/ml) to limit the analysis to permeabilized intact cells (excluding cells with sub-G1 DNA content). The γH2AX signals from these cells were analyzed using a FACS Canto (BD Biosciences).
